# Diverse Roles of the Multiple Phosphodiesterases in the Regulation of Cyclic Nucleotide Signaling in *Dictyostelium*

**DOI:** 10.3390/cells14070522

**Published:** 2025-04-01

**Authors:** Pundrik Jaiswal, Alan R. Kimmel

**Affiliations:** Laboratory of Cellular and Developmental Biology, National Institute of Diabetes and Digestive and Kidney Diseases, The National Institutes of Health, Bethesda, MD 20892, USA; pundrik.jaiswal@nih.gov

**Keywords:** cAMP, cGMP, PDEs, adenylyl cyclases, guanylyl cyclases, GPCRs, Protein kinase A, LRR/ROCO kinases

## Abstract

*Dictyostelium* is a unique model used to study the complex and interactive cyclic nucleotide signaling pathways that regulate multicellular development. *Dictyostelium* grow as individual single cells, but in the absence of nutrients, they initiate a multicellular developmental program. Central to this is secreted cAMP, a primary GPCR-response signal. Activated cAMP receptors at the cell surface direct a number of downstream signaling pathways, including synthesis of the intracellular second messengers cAMP and cGMP. These, in turn, activate a series of downstream targets that direct chemotaxis within extracellular cAMP gradients, multicellular aggregation, and, ultimately, cell-specific gene expression, morphogenesis, and cytodifferentiation. Extracellular cAMP and intracellular cAMP and cGMP exhibit rapid fluctuations in concentrations and are, thus, subject to exquisite regulation by both synthesis and degradation. The *Dictyostelium* genome encodes seven phosphodiesterases (PDEs) that degrade cyclic nucleotides to nucleotide 5’-monophosphates. Each PDE has a distinct structure, substrate specificity, regulatory input, cellular localization, and developmentally regulated expression pattern. The intra- or extra-cellular localizations and enzymatic specificities for cAMP or cGMP are essential for degradative precision at different developmental stages. We discuss the diverse PDEs, the nucleotide cyclases, and the target proteins for cAMP and cGMP in *Dictyostelium*. We further outline the major molecular, cellular, and developmental events regulated by cyclic nucleotide signaling, with emphasis on the input of each PDE and consequence of loss-of-function mutations. Finally, we relate the structures and functions of the *Dictyostelium* PDEs with those of humans and in the context of potential therapeutic understandings.

## 1. Introduction

**Figure 1:**
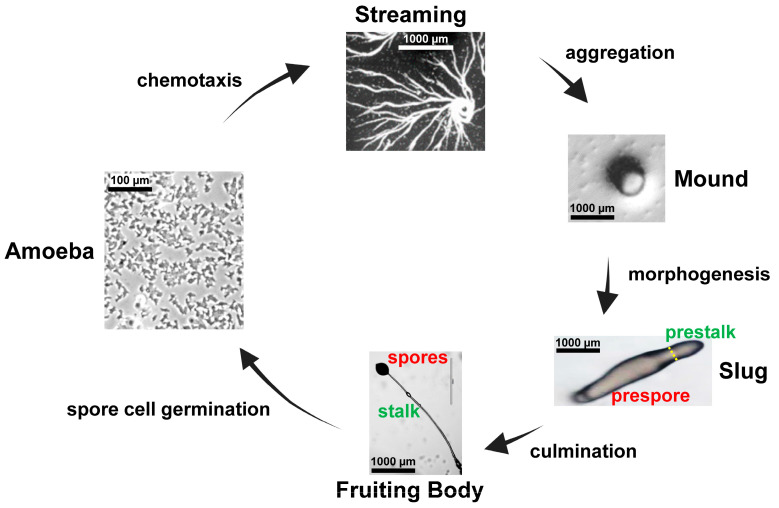
Major stages of the *Dictyostelium* developmental cycle. *Dictyostelium* grow as single amoeboid cells. When starved for nutrients, *Dictyostelium* enter a multicellular developmental program. *Dictyostelium* secrete and become chemotactic to cAMP, and streaming cells aggregate at the centers of cAMP synthesis (see discussion of Figures 8 and 9 regarding PDEs and cyclic nucleotide signaling during chemotaxis). Cells then coalesce into a tight, multicellular aggregation mound. The mound undergoes cytodifferentiation and morphogenesis into the slug, with non-terminally differentiated prestalk cells in the anterior and prespore cells at the posterior (see discussion of Figure 10 regarding PDEs and cyclic nucleotide signaling during cytodifferentiation). Culmination leads to the terminally differentiated fruiting body comprised of mature spore and stalk cells (see discussion of Figure 10 regarding PDEs and cyclic nucleotide signaling during culmination). Under the appropriate physiological conditions, spores will germinate to release amoeba for single-cell growth. Approximate size scales are indicated.

*Dictyostelium* cells grow and multiply as individual cells in nutrient-rich media. When nutrients become depleted, cells undergo a starvation response, which is mediated by the inactivation of mTORC1 and the activation of AMPK [1,2]. Nutrient depletion initiates a program of multi-cellular development. At the start, many of the genes essential for early development are upregulated, including genes involved in the regulated synthesis/degradation of cAMP and cGMP [1]. cAMP and cGMP signaling orchestrates the transition from the single-celled to the multicellular stage of *Dictyostelium* development (Figure 1), controlling chemotactic migration, aggregation, gene expression, cytodifferentiation, and morphogenesis [1,2,3,4,5].

At the onset of development, cAMP is secreted and G-protein coupled receptors for cAMP appear at the cell surface [6,7,8,9,10]. cAMP Receptor 1 (CAR1) is linked to a pathway that regulates the production of cAMP in oscillating pulse waves [4,5]. The periodic release and subsequent degradation of cAMP are crucial for establishing extracellular cAMP gradients that guide cells during chemotaxis (Figure 1). cAMP levels are tightly regulated by temporal cycling that balances synthesis and degradation [4,5,11,12]. With CAR1 stimulation, there is the simultaneous activation of an intracellular, membrane-bound adenylyl cyclase and the inactivation of an intracellular cAMP phosphodiesterase (PDE) leading to intracellular cAMP accumulation and secretion [4,12,13,14,15,16,17,18,19]. Secretion relays extracellular cAMP signaling to recruit additional cells in a coordinated response pathway.

Stimulated cells rapidly adapt to the extracellular cAMP signal. Adenylyl cyclase is inactivated, and intra- and extra-cellular cAMP are degraded by active PDEs. The entire cycle resets at a defined interval, generating cAMP waves in ~6 min pulses [4,5,12,20,21].

Other pathways, including the guanylyl cyclases, are also regulated downstream of CAR1 [22]. Accordingly, levels of intracellular cAMP and cGMP follow an oscillating pattern of accumulation [23,24] and PDE-mediated degradation [16], as do the activity patterns of their downstream cAMP and cGMP target proteins [25,26]. It is the coordinated actions of these many multiple intracellular oscillating pathways that drive the directional movement of cells within extracellular cAMP gradients and their aggregation at the centers of cAMP synthesis [4,5]. Following aggregation and multicellular formation, cells undergo fate specification and morphogenetic determination (Figure 1), also by the coordination of regulated cyclic nucleotide synthesis and degradation.

There are seven PDEs in *Dictyostelium*, each with its own unique structure, cAMP/cGMP specificity, enzymatic regulation, spatial localization, and developmentally regulated and cell-specific expression pattern [16]. The precise control of the intracellular pools of cAMP and cGMP by regulated degradation is essential to limit stimulation, maintain cellular responsiveness, and promote developmental processes.

We emphasize the distinct roles of the diverse PDEs in *Dictyostelium*, comparing their structural and functional characteristics. We integrate PDE functions with the cyclases for cAMP and cGMP syntheses, the specific cAMP and cGMP target proteins, and the intracellular and developmental pathways regulated by cAMP and cGMP signaling. Specific abnormal phenotypes resulting from inactivating mutations of individual *Dictyostelium* PDE genes underline their precise and individual roles in chemotaxis, aggregation, and development.

In humans, eleven different subfamilies of PDEs (PDE1-11) are responsible for regulating cAMP and cGMP levels [27,28]. The dysregulation of PDE activities is associated with a wide range of diseases, including cardiovascular disorders, neurodegeneracy, and cancers [27,29,30]. Understanding how PDEs regulate cyclic nucleotide signaling in both *Dictyostelium* and the more complex metazoa may offer new insights into their roles in health and disease and the potential for therapeutic intervention.

## 2. Structural Properties of PDE Members

**Figure 2:**
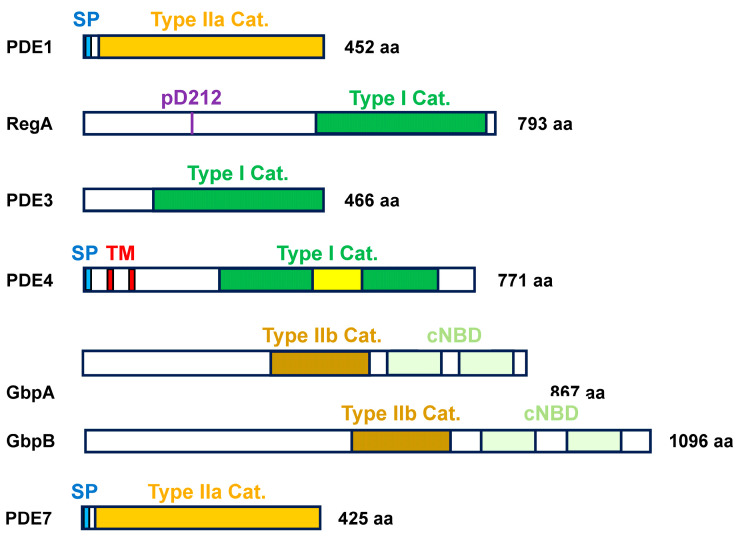
The structural domains of Dictyostelium PDEs. PDE1, PDE4, and PDE7 function to degrade cAMP in the extracellular milieu. PDE1 and PDE7 have high sequence similarity and are primarily secreted; they both may associate with the cell surface by an undefined mechanism. Their catalytic domains are part of the type IIa γ-proteobacteria family. PDE4 is a member of the type I PDE family. PDE4 is membrane-bound and modeled with two transmembrane domains and an extracellular-facing catalytic domain. Its catalytic domain is interrupted by a region of simple sequence amino acids. RegA is an intracellular type I PDE with specificity for cAMP. RegA is part of a two-component regulatory system. It is activated by a phospho-transfer from a histidine kinase to RdeA to Aspartate 212 in the response regulator receiver domain. PDE3 is an intracellular type I PDE with specificity for cGMP. GbpA and GbpB possess two cyclic nucleotide-binding domains, where binding activates their metallo-β-lactamase-type IIb PDE catalytic domain. Both are intracellular. GbpA is cGMP specific. GbpB has a preference for cAMP, but it also has a low affinity for cGMP. SP: signal peptide; TM: transmembrane domain; type IIa Cat: type IIa γ-proteobacteria catalytic family; type IIb Cat: type IIb metallo-β-lactamase catalytic family; type I Cat.: type I PDE catalytic family; cNBD: cyclic nucleotide-binding domain.

The seven phosphodiesterase (PDE) enzymes in *Dictyostelium* possess a wide range of structural and functional diversities and can be divided into two broad evolutionary class types, distinguished by the amino acid compositions of their catalytic domains (Figure 2) [16]. Class I PDEs are only present in eukaryotes and are most prevalent in mammals. Class II PDEs are primarily found in bacteria and the non-metazoa and can be further sub-classified as γ-proteobacteria- and metallo-β-lactamase-subtypes that are, respectively, type IIa and IIb PDEs; the metallo-β-lactamase features a Zn^2+^ hydrolase within its catalytic domain [16].

The various PDEs of *Dictyostelium* have distinct expression patterns, sub-cellular localizations, and specificities to cAMP or cGMP (Table 1 and Supplementary Table S1). Below is an overview of the key structural and functional characteristics among these PDEs.

### 2.1. Class I PDEs

**Figure 3:**
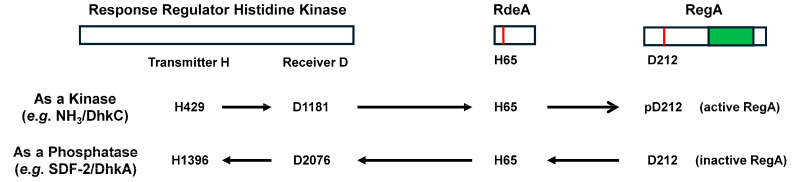
Phospho-relay regulation of RegA. Two protein components comprise phospho-relay upstream of RegA, a response regulator histidine kinase (RR-HK) and the phospho-relay RdeA; there are multiple RR-HKs that may integrate into a single RdeA. Multiple targets are likely downstream of RdeA, in addition to RegA. An active RR-HK phosphorylates an intramolecular transmitter histidine, with phospho-transfer to a receiver aspartate. This is followed by intermolecular phospho-transfer to histidine H65 in RdeA and intermolecular phospho-transfer to aspartate D212 in RegA. In a specific example, under high NH3 conditions, RR-HK DhkC is active, leading to the phosphorylation and activation of RegA. Here, intracellular cAMP levels are depleted. RR-HKs can also serve as phosphatases. SDF-2 may inhibit DhKA, with a resulting back phospho-transfer from RegA, leading to its inactivation. Here, intracellular cAMP levels are elevated. The phosphorylation sites on DhkC and DhkA are predicted, as based on comparative sequence modelling [34,36,37].

**RegA:** RegA, a class I phosphodiesterase, contains a C-terminal catalytic domain specific for cAMP hydrolysis (Figure 2) [13,14,31]. RegA has an upstream response regulatory (RR) receiver domain and functions as a part of an intracellular two-component phospho-relay system (Figure 2 and Figure 3) [14,32,33]. An activated histidine kinase (RR-HK) autophosphorylates a transmitter histidine, which undergoes intramolecular phosphotransfer to a receiver aspartate; intermolecular phosphotransfer to H65 of RdeA; and then to D212 of RegA, which leads to RegA activation (Figure 3) [14,32,34,35,36,37]. An inactive RR-HK can also function as a phosphatase, leading to the inactivation of RegA (Figure 3; Table 1) [14,32,34,35,36].

**PDE3**: PDE3, a class I phosphodiesterase, contains a 300-residue catalytic domain at its C-terminus (Figure 2), which is preceded by a region abundant with asparagine and glutamine residues. PDE3 requires a bivalent cation for activity, with a preference of Mn^2+^ [38]. The catalytic domain of PDE3 hydrolyzes cGMP into GMP (Table 1). PDE3 is intracellular and shares a structural similarity with human PDE9 [38].

**PDE4**: PDE4, a class I phosphodiesterase, is structurally modeled with two transmembrane domains and an extracellular catalytic domain (Figure 2) [39]. The catalytic domain shows high homology to human PDE8, as a conserved structure for cAMP specificity across species (Table 1) [39].

### 2.2. Class II PDEs

**PDE1 (*pdsA*) and PDE7**: PDE1, a class IIa phosphodiesterase, is secreted into the extracellular environment, but it may also associate with the external cell surface (Figure 2; Table 1). PDE1 does not share homology with PDEs in complex metazoan eukaryotes [16]. PDE1 is subject to inhibition by the secreted protein PDI [40]. PDE1 was originally identified in a genetic screen and named *pdsA* [41]. PDE7 is highly related to PDE1 and shares most of its properties (Figure 2) [16].

**GbpA and GbpB**: *GbpA* and *GbpB*, class IIb phosphodiesterases, are activated by cyclic nucleotide-binding to two domains located downstream of the metallo-β-lactamase catalytic domain (Figure 2) [16,26,42]. Both PDEs are intracellular, with GbpA exhibiting preference specificity toward cGMP and GbpB showing preference specificity toward cAMP (Table 1 and Supplementary Table S1) [16,26,42,43,44]. GbpB does have a very weak affinity for cGMP (Supplementary Table S1).

## 3. Enzymatic Properties of PDE Members

Distinct enzymatic properties of the individual PDEs can be dissected by a combination of sub-cellular fractionation, directed enzymatic inhibition, and gene-specific inactivation (Table 1 and Supplementary Table S1). As part of the Type I PDE group, RegA, PDE3, and PDE4 are collectively inhibited by 3-isobutyl-1-methylxanthine (IBMX) [16,38,39,45,46]. Caffeine has a xanthine backbone and is structurally related to IBMX [47]. However, although caffeine has been used for PDE inhibition in mammalian cells, it is avoided for PDE inhibition in *Dictyostelium*, as it has the additional effect of blocking cAMP production though the inhibition of the Adenylyl Cyclase A (ACA) activation pathway (see below) [48,49,50]. Extracellular PDE1 and PDE7 can be inhibited by 10 mM DTT [16,39,51]. Other compounds, which impact PDE activity in other systems, have not been studied as well, such as hexyl-cAMP and Rolipram [52,53].

### 3.1. Extracellular cAMP PDEs

PDE1, PDE4, and PDE7 are responsible for clearing secreted cAMP from the extracellular milieu (Table 1 and Supplementary Table S1) [16,39,41,54]. PDE1 has the highest affinity for cAMP and comprises >90% of extracellular PDE activity when extracellular cAMP concentrations are lowest (Supplementary Table S1). As extracellular cAMP concentrations rise to >1 mM, PDE4 and, to a lesser extent, PDE7 become increasingly more significant. PDE1 and PDE7 are inhibited by DTT, while PDE4 is inhibited by IBMX.

### 3.2. Intracellular cAMP PDEs

RegA and GbpB degrade intracellular cAMP (Table 1 and Supplementary Table S1) [13,14,26,31,42]. RegA has a higher affinity for cAMP and exhibits greater activity at intracellular cAMP < 1 mM; at higher intracellular cAMP concentrations, GbpB contributes increasing activity. RegA is a class I phosphodiesterase and is, thus, subject to inhibition by IBMX.

### 3.3. Intracellular cGMP PDEs

PDE3 and GbpA are the primary intracellular cGMP PDEs (Table 1 and Supplementary Table S1) [26,42]. Although GbpA has a lower affinity for cGMP than PDE3 does, under most physiological conditions, GbpA shows greater activity, generally accounting for >75% of cGMP degradation [44]. GbpB contributes some low activity (~5%) to cGMP degradation when intracellular cGMP levels are very high [46]. PDE3 is inhibited by IBMX.

## 4. Structural Properties of Adenylyl and Guanylyl Cyclases

### 4.1. The Adenylyl Cyclases

**Figure 4:**
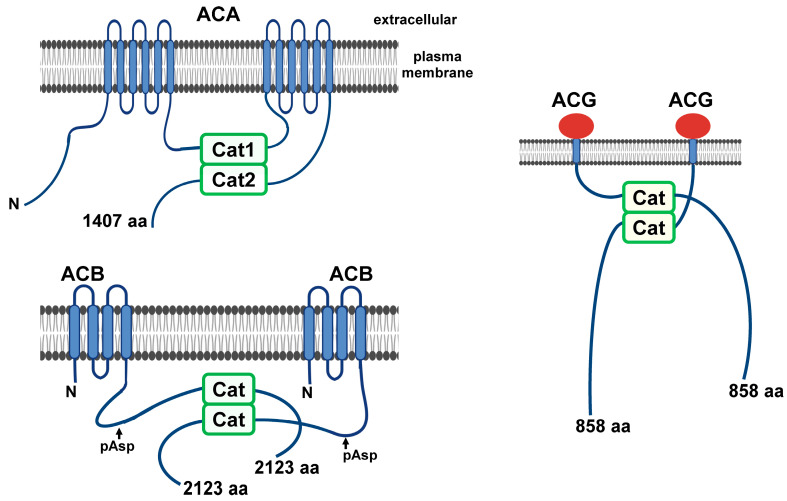
Structures of the adenylyl cyclases in Dictyostelium. ACA is a classic 12-transmembrane domain adenylyl cyclase. It possesses two intracellular catalytic domains that can be activated when dimerized. ACG is a single-pass, transmembrane protein. Two molecules are suggested to participate in catalytic domain dimerization/activation. The extracellular domain may function as a high osmotic sensor for activation. ACB was defined biochemically by enzymatic assay and as ACR by genetic cloning. ACB is a transmembrane adenylyl cyclase modeled with N- and C-terminal cytoplasmic domains; the precise topologies of the helical transmembrane domains are not defined. ACB is proposed to be part of a two-component regulatory system with activation by a phospho-transfer from an RR-HK (Figure 3). A histidine kinase (HK)-like domain is identified as N-terminal to this RR domain. However, this “HK” lacks a conserved phospho-transmitter histidine, and an active kinase function has not been confirmed. Two molecules of ACB are suggested to participate in intracellular catalytic domain dimerization/activation.

*Dictyostelium* possesses three distinct adenylyl cyclase types for converting ATP into cAMP. These are adenylyl cyclase A (ACA), adenylyl cyclase G (ACG), and adenylyl cyclase B (ACB) [19,49,55,56,57,58,59]. ACA has two separate 6-transmembrane (TM) domains, each followed by an intracellular catalytic domain (Figure 4) [19], and is structurally very similar to mammalian membrane-bound adenylyl cyclases [19]. Activity requires dimerization of those catalytic domains (Figure 4).

ACG is modeled as a single-pass TM protein with a single intracellular catalytic domain (Figure 4) [19]. By analogy to activated ACA, two ACG molecules are suggested to dimerize for catalytic activity. The extracellular domain of ACG may function as an osmosensor (Figure 4) [60].

ACB is also a TM protein with a single intracellular catalytic domain [56,57]; the exact topologies of the TM domains are not known (Figure 4). Like RegA, ACB is part of a two-component response-regulatory system. A response-receiver domain lies N-terminal to the catalytic cyclase domain, and aspartate phosphorylation is suggested to activate ACB [57,59,61]. In at least one instance (Figure 3), RdeA is not essential for the presumed phospho-relay from an RR-HK [56]. Further upstream is an RR-HK-like domain, but it lacks a predicted transmitter histidine [56]. Two ACB molecules are suggested to dimerize for catalytic activity. The nomenclature of ACB is confusing. ACB was defined both biochemically and genetically and has been alternatively called ACR/AcrA (adenylyl cyclase with a response-regulator domain) [56,58]. However, *acrA* is also used to describe the gene for the Catalase A enzyme [62,63,64].

### 4.2. The Guanylyl Cyclases

**Figure 5:**
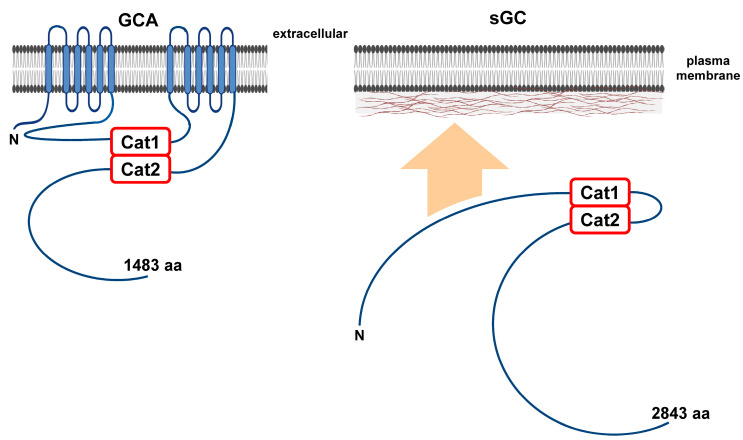
Structures of the guanylyl cyclases in Dictyostelium. GCA is a classic 12-transmembrane domain guanylyl cyclase. It possesses two intracellular catalytic domains that can be activated when dimerized. sGC is structurally analogous to soluble adenylyl cyclases found in mammalian cells. sGC possesses two intracellular catalytic domains that are active when dimerized. Still, sGC is found as both cytosolic and membrane-bound forms. The N-terminal region of sGC allows for association with cortical F-actin. sGC possesses a second function in chemotaxis that is independent of its guanylyl cyclase activity.

*Dictyostelium* has two distinct guanylyl cyclase enzymes, guanylyl cyclase A (GCA) and soluble guanylyl cyclase (sGC) [65], which catalyze the conversion of GTP to cGMP. Like ACA, GCA has 12-transmembrane regions and two intracellular catalytic domains, structurally resembling mammalian membrane-bound adenylyl cyclases (Figure 5) [22,65,66]; enzymatic activity requires the dimerization of the catalytic domains (Figure 5). sGC is structurally more analogous to mammalian soluble adenylyl cyclase than to mammalian soluble guanylyl cyclases (Figure 5) [65,66]. Although sGC is primarily cytosolic, a fraction of the enzyme localizes to the cell cortex [66], specifically translocating to the anterior of migrating cells (see below). In addition, sGC has a role in chemotaxis that is independent of its guanylyl cyclase activity [67].

## 5. Cyclic Nucleotide Target Proteins

### 5.1. cAMP Targets

**Figure 6:**
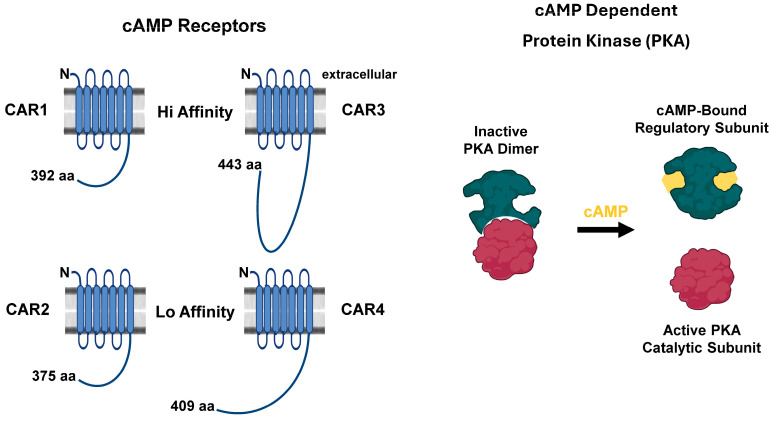
The cAMP targets in Dictyostelium. Extracellular cAMP targets four structurally similar cell surface G protein coupled receptors. cAMP receptor 1 (CAR1) has the highest affinity and is primarily responsible for regulating extracellular cAMP signaling and chemotaxis for aggregation. CAR3 also has a high affinity for cAMP and is important for prespore/spore cell fate determination; CAR4 and CAR2 have much lower affinities (>1 M) and are primarily involved in prestalk/stalk cell fate determination. Intracellular cAMP signaling is mediated by the cAMP-dependent protein kinase, PKA. PKA is comprised of a cAMP-binding regulatory subunit and a catalytic subunit. In the schematic, the regulatory and catalytic subunits are bound in the absence of cAMP, and PKA is inactive. In the presence of cAMP, the dimer dissociates, and the catalytic subunit becomes activated.

Secreted, extracellular cAMP is recognized by membrane-bound G-protein coupled cAMP receptors (CARs) (Figure 6) [6,7,8,9,10,68,69]. There are four cAMP receptors, CAR1, CAR2, CAR3, and CAR4, with different affinities for cAMP [68]. CAR1 is predominantly present on the cell surface during the early chemotaxis and aggregation stages (Figure 1), whereas CAR3 is maximal at aggregation; both have high affinity for cAMP [10,70]. CAR2 and CAR4 expression occurs at later stages during cytodifferentiation, and they have lower affinities for cAMP [71]. CAR3 and CAR4 have prominent roles in cell fate specification (Figure 1) [72,73,74,75,76].

Intracellular cAMP activates a different signaling cascade, mediated by the cAMP-dependent protein kinase (PKA; Figure 6), that is fundamentally identical to that in mammalian cells. PKA is comprised of two subunits, a cAMP-binding regulatory subunit (PKAreg) and a catalytic subunit (PKAcat). In the absence of cAMP, the regulatory and catalytic subunits are bound together in a catalytically inactive complex. In mammalian cells, two PKAreg subunits bind each other; the inactive complex is a heterotetramer of two PKAreg and two PKAcat subunits [77]. In *Dictyostelium*, the PKAreg subunits do not dimerize, and the inactive PKA is a heterodimer (Figure 6) [78]. Nonetheless, in each system, in the presence of cAMP, the regulatory and catalytic subunits dissociate, and PKAcat becomes activated (Figure 6) [78]. In *Dictyostelium*, PKA activity regulates cell–cell signaling, chemotaxis, expression of aggregation and cell fate genes, and sporulation, among other pathways (Figure 1). Cells lacking PKAcat fail to aggregate [79]. Cells lacking PKAreg have constitutively activated PKA and exhibit rapid development, in a manner that bypasses many upstream events for cAMP-dependent and -independent signaling pathways [33,78,80,81].

### 5.2. cGMP Targets

**Figure 7:**
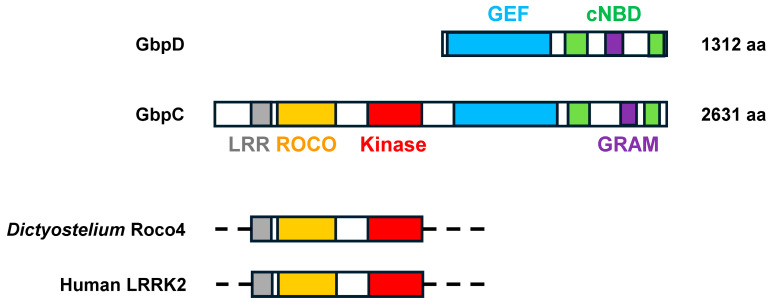
The cGMP targets in Dictyostelium. GbpC and GbpD represent the primary signaling molecules downstream of cGMP, although PDEs GbpA and GbpB are also cyclic nucleotide-binding proteins (Figure 2). Both GbpC and GbpD have GTP exchange factor (GEF) domains, which are activated by cyclic nucleotide-binding and GRAM-mediated interactions. For GbpD, Rap1 serves as the GEF target. For GpbC, the GEF targets the intramolecular bipartite, Ras-like GTPase ROCO domain for activation of the downstream intramolecular kinase and regulation of Myosin II. GbpC was one of the founding members of the LRR/ROCO kinase family, characterized by a leucine-rich repeat (LRR), a ROCO, and a kinase domain. The approximate alignments of these regions within *Dictyostelium* Roco4 and human LRRK2, relative to GbpC, are shown. Specific mutations in human LRRK2 are associated with Parkinson’s disease, and *Dictyostelium* Roco4 has proven an effective structural model [87].

GbpC and GbpD (Figure 7) bind cGMP to transduce signals further downstream. GbpC is a large multidomain protein that is nearly twice the size of GbpD. The C-terminal half of GbpC is structurally identical to full-length GbpD [26]. Both possess two cyclic nucleotide-binding domains that are separated by a GRAM domain, which facilitates membrane interaction for function [26]. Cyclic nucleotide binding activates the upstream GTP exchange factor (GEF) domain in both GbpC and GbpD.

For GbpD, the activated GEF targets Rap1 for GTP binding and activation [82]. This promotes a complex downstream pathway cascade for chemotaxis [82]. The GEF domain of GbpC targets the intramolecular ROCO (consisting of a Roc domain, Ras of complex proteins, and a Cor domain, C-terminal extension of Roc) [83,84,85]. ROCO, in turn, activates the intramolecular GbpC kinase, leading to the activation of the myosin II function in chemotactic movement (Figure 1) [26,83,85,86]. Collectively, the leucine-rich repeat (LRR), ROCO, and kinase domains at the N-terminus of GbpC define a larger protein group: the LRR kinase family (Figure 7) [83,84,85]. Significantly, mutations in one family member, human *LRRK2*, are associated with the neurodegenerative disorder Parkinson’s disease [87], and Roco4 of *Dictyostelium* is a functional model for structure/function studies of LRRK2 protein [83,84,85,88].

## 6. Good, Good, Good Oscillations

### PDEs and cAMP Oscillations

**Figure 8:**
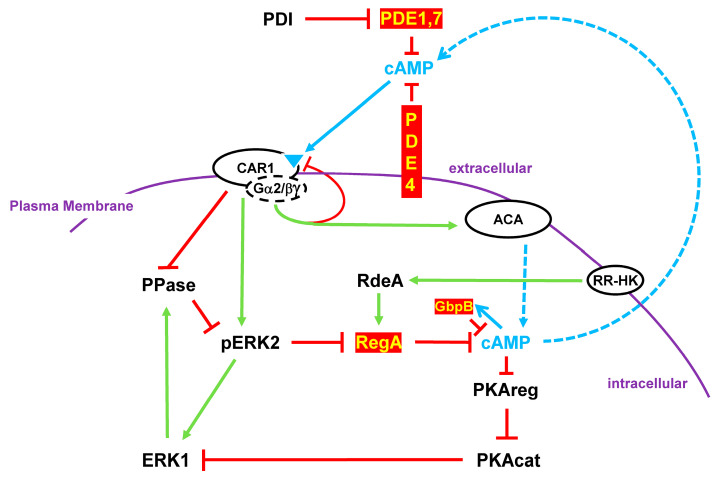
The roles of cAMP PDEs in cAMP oscillations. Quiescent, basal cells have minimal levels of intracellular cAMP, as the result of an inactive ACA and an active intracellular cAMP PDE RegA, mediated by phospho-relay from a RR-HK to RedA to RegA (Figure 3). Upon cAMP receptor (CAR1) stimulation by extracellular cAMP, ACA is activated by a complex interplay involving multiple RAS proteins, CRAC, mTORC2, and other factors. RegA is also inhibited by the phospho-activation of ERK2. Thus, intracellular cAMP accumulates. cAMP is also secreted, recruiting additional cells in response signaling. To allow cAMP levels to oscillate, stimulated cells must return to their quiescent state. This occurs in several phases. Activated CAR1 becomes de-sensitized to further stimulation. Extracellular cAMP is degraded by PDEs 1, 7, and 4; PDE1 and 7 are subject to additional regulatory inhibition by secreted PDI. Active ERK2 leads to active ERK1, promoting the de-phosphorylation and de-activation of ERK2. In the absence of CAR1 stimulation, RegA, ACA, and ERK2 return to their basal state. Finally, ERK1 is inactivated through the action of PKA. CAR1 becomes resensitized. Cells are now-re-set for another cycle of cAMP signaling. Oscillation time between on/off responses occurs with intervals of ~6 min.

For multicellular development, individual *Dictyostelium* cells must migrate toward a common, collective center for aggregate formation (Figure 1). Within a starved, developmentally responsive cell population, centers become established that initiate cAMP signal production. Proximate cells respond by moving inwardly toward the cAMP signaling centers and by further secreting cAMP outwardly to recruit additional more distal cells for a collective chemotactic cell stream. Cells oscillate between responsive and non-responsive (adapted) states, at ~6 min intervals [4,5,20,21]. This ensures that there is a persistent pulse of a directionally outward flow of a cAMP signaling gradient, with a consequent inward cell movement and multicellular aggregation (Figure 1). The production of these cAMP oscillations requires the coordinated regulation of cAMP synthesis as well as PDE-mediated cAMP degradation (Figure 8) [4].

Unstimulated cells have inactive membrane-bound ACA and active intracellular RegA PDE; intracellular cAMP levels are minimal [11,13,14,15,16,17,18,19]. Upon the stimulation of cAMP receptor CAR1, both ACA and ERK2 are activated. ACA drives intracellular cAMP synthesis, while phospho-activated pERK2 inhibits RegA [4,12,13,21]. Intracellular cAMP accumulates and is secreted, recruiting other cells for signal response (Figure 8). Activated CAR1 promotes a series of additional downstream pathways that are required for regulated gene expression and for chemotaxis (see below). Cells migrate collectively toward cAMP signaling centers, where they form multicellular structures.

Activated CAR1 also becomes de-sensitized/adapted to a saturating or non-varying concentration of cAMP [4]. As a result, ACA activation ceases, ERK2 activation promotes its own de-phosphorylation/inactivation mediated by ERK1, and RegA is re-activated. In addition, all other activation response pathways downstream of CAR1 also adapt. This arrests chemotaxis [4].

With the inactivation of ACA and the activity of RegA (and GbpB), intracellular cAMP levels are degraded, and cAMP secretion is halted. Extracellularly, PDE1, PDE4, and PDE7 clear cAMP, removing the CAR1 stimulus. Cells are, thus, returned to a basal, but an unresponsive, state (Figure 8) [4,12].

With a temporal delay, CAR1 de-adapts and becomes re-responsive. Intracellular cAMP activates PKA (Figure 8), which inactivates ERK1, allowing for the re-activation of ERK2. With the decline in intracellular cAMP, PKA is inactivated. Cells are, thus, again primed for another cycle of cAMP response, cAMP secretion, and directional chemotaxis. Oscillations occur at ~6 min intervals (Figure 8) [4].

Loss-of-function studies have revealed a developmental complexity to the essential nature of individual PDEs. *regA*-null cells secrete cAMP, but signaling waves do not propagate normally, streaming is disordered, and aggregate sizes are small [89]. Chemotactic cell-shape patterns are also disrupted (see below) [89]. The elevated activity of GbpB is not sufficient to compensate for inactive RegA (Supplementary Table S1). In the absence of RegA, intracellular cAMP levels are increased [21], and PKA-dependent cell-specific gene expression can still proceed [78].

PDE1 controls extracellular cAMP levels during cell streaming, but it also does so at aggregation and during cytodifferentiation (Figure 1). Extracellular cAMP concentrations are maximal at the later development times, and PDE4 and PDE7 play an increasingly significant role (Supplementary Table S1) [16,39,41,54]. Multiple PDE1 promoters are required to regulate both expression level and cell specificity during development [90,91]. As with loss of RegA, the disruption of normal cAMP signaling in either *pde1*-null or *pde4*-null cells leads to abnormal development [39,41].

ACA is required throughout the *Dictyostelium* developmental cycle (Figure 1); however, it is most predominantly expressed during early aggregation. Cells lacking ACA cannot generate an extracellular cAMP signaling pattern and fail to aggregate [19,92]. However, they are responsive to cAMP and will aggregate (i.e., synergize) in the presence of cAMP waves secreted by wild-type cells [19]. The low level expression of ACB and ACG can contribute necessary intracellular cAMP levels in the absence of ACA.

## 7. Turn, Turn, Turn

### PDEs and Chemotaxis

The oscillating pulses of cAMP also direct cell movement (Figure 1). Specific cellular proteins and factors associate with the anterior or posterior of the cell, which enhances cell extension and pseudopod formation at the leading edge in the direction of the gradient and suppresses lateral and rear pseudopod formation. Cells, thus, orient, polarize, and migrate (and turn) directionally toward the gradient flow.

**Figure 9:**
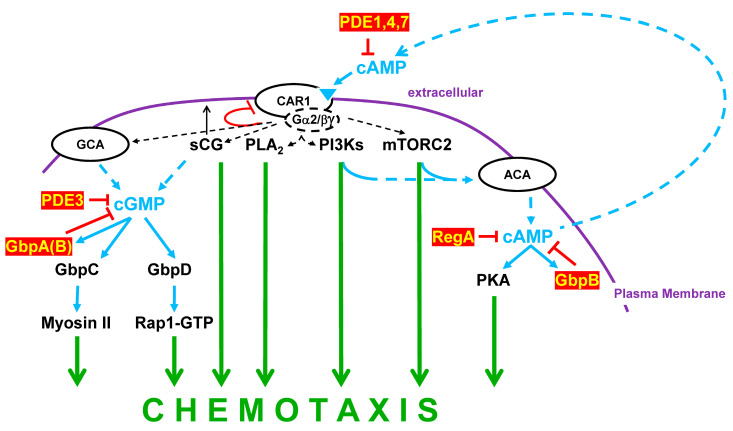
The Roles of PDEs in chemotaxis. Multiple pathway arms are activated downstream of cAMP receptor signaling, which collectively direct chemotaxis during developmental aggregation. The primary cyclases activated downstream of CAR1 during aggregation are GCA, sGC, and ACA. GCA and sGC cooperate to promote cGMP accumulation. cGMP activates the GEF domains in proteins GbpC and GbpD. The GbpD GEF domain promotes Rap1-GTP formation, with the consequent pathway signaling driving chemotaxis. The GbpC GEF domain then activates the intramolecular ROCO domain (Figure 7), whose target is the GbpC kinase in the myosin II pathway. cGMP will also activate GbpA, which, in conjunction with PDE3, degrades intracellular cGMP. GbpB plays an additional minor role in cGMP degradation. ACA promotes cAMP accumulation, with the primary intracellular target PKA (Figure 6). cAMP will also activate GbpB, which, in conjunction with RegA, degrades intracellular cAMP. Fundamentally, chemotaxis is dependent on the collective signaling pathways downstream of CAR1, which regulate and coordinate cellular structural parameters that drive directional movement within a cAMP gradient. Other functions proximate to CAR1, but independent of cyclic nucleotide signaling, include PI3Ks, mTORC2, PLC, PLA2, and the non-catalytic activity of sGC. Many are immediately responsive to CAR1-dependent RAS activations, downstream of G protein signaling. It should also be evident that, with the oscillation of extracellular cAMP and the on/off cycling of CAR1 activation (Figure 8), intracellular levels of cAMP and cGMP, and other downstream pathway activities, will also oscillate. This serves to re-enforce directed cell migration toward the highest concentrations of cAMP.

CAR1 stimulation activates not only the cAMP signaling pathway (Figure 8), but also multiple other circuits (e.g., PLC, PLA_2_, PI3K, mTORC2; Figure 9), which collectively integrate for the cytoskeletal reorganizations essential for directional chemotaxis [93,94,95].

Guanylyl cyclases GCA and sGC are other CAR1-mediated regulatory arms for actin/myosin II/cytoskeletal targeting; cGMP homeostasis is further regulated by cGMP phosphodiesterases PDE3 and GbpA, with very minor input from GbpB (Figure 2 and Figure 9; Table 1; and Supplementary Table S1).

There are two pathways downstream of cGMP signaling that involve binding proteins GbpC and GbpD (Figure 7 and Figure 9). Both are cGMP-activated guanine nucleotide exchange factor (GEF) proteins. The GbpD GEF promotes Rap1-GTP formation and downstream-dependent functions [82]. GbpC GEF functions through its own ROCO to activate an intramolecular kinase that functions through myosin II to suppresses pseudopod formation at the rear of the cell [85]. *gbpC*-null cells show defective chemotaxis in response to an extracellular cAMP gradient due to an inability to polarize [26,86]. *gbpD*-null cells exhibit an opposite phenotype, with strong chemotaxis and hyperpolarization. Apart from cyclase activity, sGC translocates to the anterior cell cortex, where it functions by refining pseudopod formation at the leading edge of chemotaxing cells (Figure 5 and Figure 9) [67].

## 8. Instant Karma

### RegA and Fate Specificity

As multicellular aggregates form, two broad fate choice specificities are initiated, giving rise to ~20% prestalk and ~80% prespore cells (Figure 1). These are the non-terminally differentiated precursors to the terminally differentiated stalk and spore cells (Figure 1). Morphogenetically, as the prestalk and prespore cells differentiate within the aggregation mound, they also begin to sort. Prestalk cells move to the top of the mound and extend to the tip. Prespore cells form the mound base. The tipped mound extends into a migrating pseudoplasmodium/slug, with prestalk cells at the anterior and prespore cells at the posterior (Figure 1). However, there is significant complexity, with sub-types of each population forming and sorting separately. In addition, the populations are not fully committed and can transduce between themselves under defined conditions. At culmination, the slug transits to the terminally differentiated fruiting body (Figure 1), comprised of spore and stalk cells, again with sub populations.

**Figure 10:**
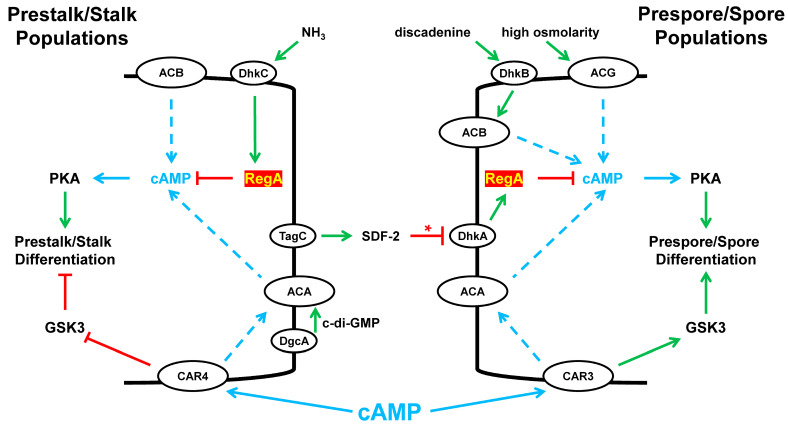
The role of RegA in cell-fate specification. Prestalk/stalk and prespore/spore differentiations are complex and cannot simply be described as a binary choice. There are subtypes within each major class, but cell development is also a continuum, from precursor to terminal differentiation. Nonetheless, prestalk/stalk and prespore/spore differentiations are stylized and compressed into a single representation for each. Prespore cells express CAR3, and prestalk cells express CAR4. In multicellular structures, extracellular cAMP-stimulated CAR3 drives PKA (via ACA) and GSK3 activations, which are required for prespore differentiation; extracellular cAMP-stimulated CAR4 drives PKA (via ACA) activation but inhibits GSK3, as required for prestalk differentiation. Prespore and prestalk cells are mostly sorted from one another. First, prespore cells form the base of the aggregation mound. The prestalk cells extend to the tip of the mound and then further to an elongated migrating slug. In general, within the slug, prestalk cells form the anterior 20%, and prespore cells the posterior 80%. However, there are subtypes, substructures, and even intermingled cell-types that are not further detailed. Morphogenetic and cell developmental progression for culmination beyond the slug stage requires continued intracellular cAMP accumulation in pretalk cells. Environmental NH3 is one factor that suppresses cAMP accumulation. RR-HK DhkC becomes activated, which maintains the high phospho-activity of RegA, which limits cAMP accumulation, culmination, and terminal differentiation of both the stalk and spores. Whence the slug moves to the open area where NH3 is dispersed, RegA activation is attenuated. Several processes then facilitate intracellular accumulation in prestalk and prespore cells. Limited RegA activity, coupled with the actions of ACA and ACB, leads to an increase in intracellular cAMP, which promotes culmination. Prestalk lineage cells produce two factors that further enhance cAMP levels. DgcA is a dinucleotide cyclase, and c-di-GMP is a presumed activator of ACA in prestalk cells. TagC in prestalk cells mediates the release of SDF-2, which inhibits RegA in prespore cells. Functionally, SDF-2 acts to de-phosphorylate/inactivate RegA. In the presence of SDF-2, the RR-HK DhkA is a phosphatase of RegA (Figure 3), although the precise mechanism is not determined. This is depicted as HK inhibition (*). Two other inputs promote intracellular cAMP accumulation. Discadenine functions through RR-HK DhkB to activate ACB, independently of RdeA (Figure 3 and Figure 4). High osmolarity activates ACG (Figure 4). Interactions of NH3, c-di-GMP, and discadenine with their target proteins are not meant to indicate direct binding. The mechanistic interactions are not known.

Intra- and extra-cellular cyclic nucleotide signaling continues to be essential to all phases of cytodifferentiation and morphogenesis (Figure 10). To be clear, events are only diagrammatically presented and are compressed into a single time representation, with both precursor and terminal cells collectivized as a single cell-type. As illustrated, we highlighted only some essential signaling centered through the control of PDE RegA function in fate decisions and discussed in the context to a single principal control point (Figure 10).

Early steps to cytodifferentiation are determined by the expression of cell-specific markers within the undifferentiated population. Regarding extracellular cAMP response, CAR3 is expressed in cells of the prespore path and CAR4 in cells of the prestalk path [71,72,73,74,75,76,96]. The stimulation of each receptor leads to the activation of ACA, the accumulation of intracellular cAMP, and the activation of PKA. PKA is essential for the differentiation of both cell types [97]. Polarized differentiation relies on differences from the activity of GSK3 [73,74]. CAR3 promotes GSK3 activity for prespore differentiation, whereas CAR4 inhibits GSK3 for prestalk differentiation (Figure 10) [72,73,74,75,76]. High-level intracellular cAMP accumulation and the activity of PKA are required for continued prestalk/prespore differentiation and slug stage development [78]. In addition, there are essential intercommunication signals transmitted between the prestalk/prespore populations. These coordinate morphogenesis with terminal cell-type progression, but they also serve to maintain cell-type ratios.

Culmination is the morphogenetic process between the slug and fruiting body formation (Figure 1). During culmination, the stalk and spore cells become terminally differentiated. However, culmination requires elevated intracellular cAMP, an input which is suppressed by activated RegA (Figure 10). In the wild, migrating slugs may be surrounded in a dense undergrowth. Culmination in this milieu is not developmentally advantageous and is inhibited. Accumulated environmental NH_3_ promotes RR-HK DhkA activity (Figure 3) [37], which maintains an active RegA, limits intracellular cAMP, and prevents culmination (Figure 10) and which suppresses the progression of terminal cytodifferentiation [37].

Slugs in a more open locale are no longer exposed to environmental NH_3_ cues, and the phospho-mediated activity of RegA becomes limited. Prestalk cells also express DgcA, a di-cyclic GMP synthase; di-c-GMP is presumed to further activate ACA [98]. Additional cAMP is suggested to arise from ACB (Figure 10).

Arrested slugs are also blocked for sporulation, terminal spore differentiation (Figure 1). Developing prestalk cells secrete a factor(s) that induces prespore and spore differentiation [99,100].

During culmination (Figure 1), the coordinated interactions of prestalk/prespore cells, with the requirement of TagC in prestalk cells, release the prespore-specific RegA inhibitor SDF-2 (Figure 10) [34,35,101,102]. SDF-2 interacts with the RR-HK DhkA [34] that can then act as a functional phosphatase of RegA (Figure 3) [34]. The precise mechanism is not described, but for consistency, SDF-2 actions are schematically described as an inhibitor of DhkA, permitting phospho-transfer away from RegA and inhibition. Regardless, the inhibition of RegA induces PKA. ACB and ACG provide other intracellular cAMP inputs [19,56,60]. The precise timing of RegA inhibition is critical, as *regA*-mutant cells exhibit premature sporulation. ACB and ACG provide other sources for intracellular cAMP accumulation, which is required for sporulation [19,56,60]. For ACB, activation is suggested to require an RdeA-independent phospho-transfer from the RR-HK DhkB, with input from discadenine [61]. Cells lacking ACB exhibit defects in terminal spore differentiation [56]. Finally, the germination of spores to growth-phase cells is inhibited by highly active ACB and ACG. Thus, discadenine and elevated osmo-concentrations block germination.

## 9. This Is the End

### PDEs, Disease, Therapeutics, and Drug Discovery

*Dictyostelium* has proven a particularly valuable model for the study of fundamental cellular and developmental processes and their foundational associations with many human diseases [103,104,105,106,107,108,109,110,111,112,113,114,115,116,117,118,119,120,121,122,123]. Such studies have addressed pathogenic infection, cellular overgrowth, and neurological disorders of Alzheimer’s and Huntington’s Disease, among others. These few examples are presented to emphasize how studies in *Dictyostelium* can be applied to understand novel aspects of global regulatory pathways, which may provide new insights to human genetic disorders and, perhaps, offer innovative approaches for treatment.

Phosphodiesterases (PDEs) represent a specialized enzymatic system used to regulate cyclic nucleotide signaling throughout development, in all cell types, and in organisms as diverged as *Dictyostelium* and humans. The interactions among the different PDE classes, with their unique structural characteristics, enzymatic specificities, and differential expressions, underscore the molecular complexity of cyclic nucleotide signaling that orchestrates sophisticated cellular behaviors.

Human PDEs are classified into 11 different gene families. However, there are 21 gene subtypes, in addition to alternative mRNA splice variants, that collectively generate > 100 PDE protein species [27,28,29,124,125,126].

As in *Dictyostelium*, human PDE subtypes may have specificity for cAMP (PDE 4, 7, and 8) or for cGMP (PDE 5, 6, and 9) [29]. Some exhibit regulatory sensitivity to cyclic nucleotide input. Significantly, human diseases and complex disorders have been specifically associated with abnormal PDE function. Mostly, the connection is to PDE hyperactivity (i.e., reduced cyclic nucleotide levels), leading to a focus on the development of selective PDE inhibitors.

Human PDE9 is expressed in the heart, kidneys, and brain, where it functions to degrade cGMP. Low cGMP levels have been observed in patients with cardiac failure or Alzheimer’s disease. Increasing cGMP concentrations by inhibiting PDE9 activity has been a therapeutic strategy for elevating cGMP patient levels and improving diastolic heart function or enhancing cognitive ability in Alzheimer’s patients [127]. A treatment parallel has been observed with PDE8. Human PDE8 variants PDE8A and PDE8B are expressed in immune cells, lung, kidney, testis, and thyroid. The targeted inhibition of PDE8 has proven an effective approach for respiratory conditions, such as asthma and chronic obstructive pulmonary disease (COPD), inflammatory and autoimmune disorders, and possibly breast cancer and cardiac pathologies [128,129,130]. Drugs that target specific PDEs for other conditions are also approved, with the potential for more broad therapeutic benefits to metabolic syndromes, cancers, autoimmune diseases, and neurological disorders. New drugs, drug screens, and other interventions for PDE functions are imperative [27,28,29,124,125,126].

The utility of novel systems has often provided innovative approaches to novel drug discoveries. Interestingly, human PDEs, PDE8 and PDE9, exhibit a unique sequence relationship with *Dictyostelium* PDE4 and PDE3, respectively, which may serve as functional surrogates [38,39]. Both HsPDE8 and DdPDE4 are cAMP-specific, with shared pre-catalytic and catalytic domains [39]. Both HsPDE9 and DdPDE3 target intracellular cGMP pools [29,38,127]. Indeed, HsPDE9 is more closely related in sequence to DdPDE3 than to any other human PDE [38]. Still, we recognize that the *Dictyostelium* PDEs are not perfect homologs to the human. Neither HsPDE8 nor HsPDE9 are inhibited by IBMX [28], whereas both DdPDE4 and DdPDE3 are IBMX-sensitive. Alternatively, gene substitution/replacement strategies may allow a distinct approach to assess human PDE functions and inhibitions.

Finally, *Dictyostelium* cGMP-binding protein GbpC belongs to the LRR kinase family. The human LRRK2 kinase is associated with Parkinson’s disease; mutations in the *LRRK2* gene increase the risk of Parkinson’s disease, and the inhibition of kinase can help control the condition [87]. Since LRRK2 is a drug target for treating Parkinson’s disease, studying *Dictyostelium* GbpC and related LRR/ROCO kinases in *Dictyostelium* for potential drug discovery may provide significant insights into Parkinson’s therapeutics.

## Figures and Tables

**Table 1:** The Multiple PDEs of Dictyostelium.

PDE	Class Type	Localization	Substrate	Pharmacological Inhibition	Cellular Activation	Cellular Inhibition
PDE1 (PsdA)	IIa ^ γ-proteobacteria	Extracellular	cAMP (cGMP *)	DTT	**-**	PDI
RegA	I	Cytosol	cAMP	IBMX	Response Regulator	Activated ERK2
PDE3	I	Cytosol	cGMP	IBMX	**-**	**-**
PDE4	I	Extracellular Facing Membrane-bound	cAMP	IBMX	**-**	**-**
GbpA	IIb ^ metallo-β-lactamase	Cytosol	cGMP	**-**	cGMP	**-**
GbpB	IIb ^ metallo-β-lactamase	Cytosol	cAMP cGMP	**-**	cAMP cGMP	**-**
PDE7	IIa ^ γ-proteobacteria	Extracellular	cAMP (cGMP *)	DTT	**-**	(PDI)

^ The catalytic domains of Class II PDEs can be further divided into two phylogenetic sequence relationships. * While these extracellular PDEs have in vitro activity toward cGMP, cAMP is the primary extracellular cyclic nucleotide pool.

## Data Availability

Not applicable.

## Supplementary Materials

The following supporting information can be downloaded at: https://www.mdpi.com/article/10.3390/cells14070522/s1, Supplementary Table S1: Activity Patterns of *Dictyostelium* PDEs, Relative to in vivo [cNMP].
