# Supplementary material for: Diverse Roles of the Multiple Phosphodiesterases in the Regulation of Cyclic Nucleotide Signaling in Dictyostelium
Source: Cells. 2025 Apr 1;14(7):522. doi: 10.3390/cells14070522 (PMC11988041; doi:10.3390/cells14070522)
Supplement: Supplementary file 1 [file cells-14-00522-s001.zip › cells-3516191-supplementary.pdf]

SUPPLEMENTAL TABLE SI

Activity Patterns of *Dictyostelium* PDEs, Relative to *in vivo* [cNMP]

PDE	Affinity Type (K_m)	Relative Activity to Extracellular cAMP, Aggregation (at <1 mM)	Relative Activity to Extracellular cAMP, Multicellular (at >5 mM	Relative Activity to Intracellular cAMP, Aggregation	Relative Activity to Intracellular cGMP, Aggregation (at 5 mM)
PDE1 (PsdA)	Hi (<1 mM)	>90%	<45%	-	-
RegA	Hi (<5 mM)	-	-	>55%, at <1 μM	-
PDE3	Hi (<1 mM)	-	-	<20%, at >10 μM	<25%
PDE4	Lo (>10 mM)	<5%	>45%	-	(50%, at <0.01 μM)
GbpA	Lo	-	-	-	-
GbpB	Lo	-	-	-	>75%
PDE7	Lo (>10 mM)	<5%	<5%	<45%, at <1 μM	<10%
